# Burden of Vaccine-Preventable Diseases in People Living with HIV

**DOI:** 10.3390/vaccines12070780

**Published:** 2024-07-16

**Authors:** Hady Samaha, Arda Yigitkanli, Amal Naji, Bahaa Kazzi, Ralph Tanios, Serena Maria Dib, Ighovwerha Ofotokun, Nadine Rouphael

**Affiliations:** 1 The Hope Clinic of the Emory Vaccine Center, Division of Infectious Diseases, Department of Medicine, Emory University, Decatur, GA 30030, USA; ayigitkanli3@gatech.edu (A.Y.); amal.naji@piedmont.org (A.N.); bahaa.kazzi@emory.edu (B.K.); ralph.tanios@emory.edu (R.T.); serena.maria.dib@emory.edu (S.M.D.); nroupha@emory.edu (N.R.); 2 Division of Infectious Diseases, Department of Medicine, Emory University, Atlanta, GA 30322, USA; iofotok@emory.edu

**Keywords:** HIV, vaccines, vaccine-preventable diseases, burden, barriers

## Abstract

Vaccine-preventable diseases (VPDs) pose a serious public health concern for people living with HIV (PLH). PLH experience a delayed and weakened response to many vaccines available, compared to the general population. Lower seroconversion rates, along with a decreased efficacy and durability of vaccines, increases the susceptibility of PLH to VPDs. Vaccination guidelines specifically targeting this population have been modified to overcome these challenges. However, vaccine uptake remains suboptimal due to multiple barriers, highlighting the need for further studies and the additional implementation of public health measures specifically tailored to PLH.

## 1. Introduction

The immunodeficiency caused by HIV renders the host more susceptible to infectious diseases, including vaccine-preventable diseases (VPDs), with worse outcomes [1,2]. Studies have shown that, despite adequate therapy with ART, people living with HIV (PLH) continue to have a higher risk of infection with VPDs than the general population. While many vaccines are recommended for PLH, the burden of VPDs [3,4,5] remains substantial due to suboptimal responses to vaccination or less-than-satisfactory rates of vaccine coverage [6,7,8].

## 2. Common Vaccine-Preventable Diseases in People Living with HIV

### 2.1. Influenza

Influenza infection is more common in PLH, leading to more acute and prolonged symptoms and increased risks of complications with higher mortality rates [9,10,11,12]. While recommendation for influenza vaccine is universal, PLH should receive an annual seasonal inactivated influenza vaccine and a not live attenuated influenza vaccine. Although multiple studies have shown no increased adverse events in PLH receiving the live attenuated seasonal influenza vaccine [13,14], a concern over prolonged shedding persists; therefore, this intranasal vaccine is not recommended for PLH.

Cohen et al. showed that incidence of influenza infection in PLH was four to eight times higher than uninfected individuals. When comparing three consecutive influenza seasons between 1991 and 1994, Lin et al. showed that PLH had excess death rates, with 19.74, 15.38, and 10.17 deaths per 10,000 persons (vs. rates of 1.40, 1.62, and 1.48 for the general US population who were 13 years and older) [10].

Data on influenza vaccination rates in PLH are limited. In a recent study using data extracted from Kaiser Permanente North California electronic health records, Imp et al. found an almost 50% higher rate of influenza vaccination in PLH than in uninfected individuals, although the study might be limited by a sampling bias [15].

Studies on influenza vaccination in PLH show that the vaccine is safe and well tolerated [16,17]. Additionally, seroconversion and seroprotection rates varied by vaccination strategy, population studied, and viral strain [18]. When comparing antibody levels post vaccination, PLH were significantly less likely to generate an antibody response despite a high CD4 cell count and receipt of antiretroviral drugs when compared to uninfected individuals [19]. The host’s immune status and matching to circulating strains significantly impacted the response to the influenza vaccine with an efficacy ranging between 27 and 78% in PLH depending on the influenza season [20].

Different strategies were explored to enhance vaccine immunogenicity and effectiveness in PLH. Adjuvanted influenza vaccines, such as the MF-59 adjuvant mixed with the inactivated seasonal influenza vaccines, were shown to lead to higher antibody levels [21] compared to non-adjuvanted formulations. Moreover, vaccines that contain a higher amount of hemagglutinin, designated as high-dose influenza vaccines, were more successful in limiting influenza in high-risk populations compared to standard-dose vaccines [22,23]. However, Nunes et al. reported that even when the seasonal influenza vaccine was given at a higher dose in pregnant women with HIV, it was still unable to produce similar immunogenicity to a standard-dose vaccine in pregnant women without HIV [24]. Intradermal influenza vaccination represents another viable strategy to enhance vaccine response in PLH by utilizing the skin, the largest immunologic organ. However, Seo et al. reported comparable immunogenicity between intradermal and intramuscular influenza vaccines in PLH [25].

### 2.2. Pneumococcal Disease

*Streptococcus pneumonia* is a widespread cause of infection and is known to be the most common cause of acute otitis media, sinusitis, pneumonia, and meningitis [26].

Pneumococcal vaccination is recommended in all PLH. Two vaccines, the PCV (pneumococcal conjugate vaccine) and PPSV (pneumococcal polysaccharide vaccine), are currently recommended to protect against infection. The vaccine formulation depends on the patient’s prior vaccination status against pneumococcal pneumonia. Patients without a history of pneumococcal vaccination should receive either PCV 20 or PCV 15. If they receive the latter, it is recommended to receive a PPSV23 booster at least 8 weeks later [27]. For other patients, recommendations for pneumococcal vaccines are based on age or the presence of risk factors that increase the likelihood of invasive pneumococcal disease (IPD).

PLH have a significantly higher burden of disease due to pneumococcal disease compared to their non-infected counterparts. Madhi et al. demonstrated that PLH have a higher prevalence of *Streptococcus pneumoniae* and *Haemophilus influenzae* colonization compared to people without HIV [28]. This association between HIV and pneumococcal disease is further confirmed by the fact that the highest burden of pneumococcal disease is observed in low-income countries where HIV is more prevalent [29]. Furthermore, *S. pneumoniae* is the leading bacterial opportunistic infection and has a high rate of recurrence among PLH [30]. Evidence indicates that PLH are prone to substantially greater rates of IPD than uninfected individuals [31,32,33]. A retrospective study on IPD in PLH in San Francisco reported a substantially greater occurrence of IPD than uninfected individuals prior to the availability of potent antiretroviral treatments [31]. Similarly, a meta-analysis by van Aalst et al. showed a pooled incidence of IPD of 331/100,000 person years in PLH in the HAART era in non-African countries, 318/100,000 in African countries, and 10/100,000 in healthy control cohorts [33].

The efficacy of the pneumococcal vaccine was highlighted by French et al. who conducted a randomized, double-blind trial of the 7-valent PCV, showing 74% efficacy in PLH despite low CD4 counts [34]. Studies indicate that a combination of the two available vaccine formulations leads to considerably higher immunity [35,36], although more studies are needed to evaluate the long-term immunogenicity of this vaccination strategy and test high valency vaccines in PLH.

### 2.3. Meningococcal Disease

It is currently recommended that all PLH aged ≥ 2 months receive the meningococcal conjugate vaccine. Previously unvaccinated individuals aged 2 years or older should receive a two-dose primary series followed by a booster 8 weeks later. Additional booster doses should be administered 3 years later (if the last booster was given before age 7) or at 5-year intervals (if the most recent booster dose was after age 7) [37].

During the period of 2017–2021, 15 meningococcal cases were reported each year among PLH in the US, 1.5–4.3% of total annual meningococcal cases [38]. In 2022, this number increased to 29 cases (9.8% of all cases), with 22 of those cases being unvaccinated persons. In terms of commonalities among the cases, nine were Black or African American persons, and seven were among men who had sex with men (MSM). The increased risk of infections was also coupled with a low rate of vaccination for PLH, with a survey in 2016 showing only 16.3% of PLH having received at least one dose of MenACWY within 2 years after diagnosis.

Vaccine efficacy for PLH is slightly lower than those uninfected with HIV, at 81%. Immune response was also lower for the serogroups (seroconversion to A: 68%; C: 52%; Y: 73%; and W-135: 63%), especially in the setting of advanced HIV [39].

### 2.4. Haemophilus Influenzae

*Haemophiles influenzae type b* (Hib) is a life-threatening infection for those with immunocompromising conditions, including PLH, predominantly targeting children.

Monovalent vaccination for Hib is administered in three doses for infants ages 2–6 months, with an additional booster dose given at age 12 months [40]. The CDC recommendation for the Hib vaccine is for all between 12 months to 5 years of age for individuals without HIV, with vaccination recommended for all PLH from 2 months of age and later [41].

There are an estimated 8.1 million annual episodes of serious illness and 371,000 childhood deaths as a result of Hib, 8100 of which were among children living with HIV [42]. The mortality is worsened by the increased risk for Hib-related bacteremic pneumonia, which is 20 times more common in PLH [43]. The meningitis rate is only slightly higher, but the severity of neurological sequelae in those infected was 71%, compared to 33% for children living with HIV [42]. Cases of Hib pneumonia have been described in children with HIV fully vaccinated against Hib, with a vaccine efficacy as low as 44%.

A vaccine efficacy of 100% has been reported in preventing Hib meningitis [43].

### 2.5. Hepatitis A

The CDC recommends a hepatitis A vaccination schedule for PLH consisting of two vaccine doses within 6–18 months apart, regardless of CD4 count [44]. However, at 1 month post last vaccination, an IgG antibody test should be obtained [45]. When combined with hepatitis B vaccination, it is a total of three doses.

PLH with an underlying liver condition are at risk for severe disease from hepatitis A virus (HAV) infection. In a HAV outbreak in 2019, out of 194 cases, 119 were hospitalized (including 4 PLH) and 2 died [46]. Susceptibility among unvaccinated people, aged 20 years or older, in the US is 74.1%, with an increase in HAV infection, from 1239 annual cases in the US in 2014 to 2019 annual cases between 2016 and 2018 [46]. In a study conducted in 1999 following 800 PLH, the incidence rate of hepatitis A was estimated to be 5.8% per year [47]. The population of PLH that possesses evidence of HAV immunity is 64%, with the remaining third not receiving HAV vaccination [48].

HAV vaccination provides early seroconversion in the general population, with over 90% one month after vaccination [49]. Yin-Lin et al. conducted a prospective study during the HAV outbreak in Taiwan. They included 1553 PLH, with 1001 having received at least one dose of the HAV vaccine, and 965 having received two vaccine doses 6 months apart. It revealed that less than 8% achieved seroconversion at 4 weeks, with an overall seroconversion rate of 41% before the second vaccine. The rate increased to 94% at weeks 28–36 in the per protocol analysis. In this study, outcomes were better in younger patients with undetectable HIV RNA levels [50]. The impaired HAV vaccine immunogenicity was further demonstrated in the clinical trial completed by Launay et al., which compared a three-dose vs. two-dose HAV vaccine schedule. The seroconversion rate was only 72% at 28 weeks in the two-dose schedule. The three-dose schedule provided significantly higher HAV antibody titres [51]. A study conducted by Crum-Cianflone et al. showed that 85% of vaccinated PLH had a durable vaccination response to HAV lasting 6 to 10 years [52]. For individuals without HIV, the seroprotection of 98.5% of Hep A vaccinations has been shown to last for 10 years [53].

### 2.6. Hepatitis B

The hepatitis B virus (HBV) can be transmitted either vertically (perinatal transmission), horizontally, sexually, or parenterally.

Many infectious diseases and HIV-related societies recommend vaccination with a high-dose HBV vaccine for all adults and adolescents living with HIV. An ultra-rapid vaccination schedule should be considered for patients with CD4 > 500 cell/mm^3^ if adherence to a full course is doubtful. Typical vaccination schedules have shown to provide insufficient protection for PLH [54,55,56]. If post-vaccination concentration is not ≥10 mIU/mL, a second three-dose series of HBV is recommended [3,5].

HBV infection rates are greater in PLH compared to the general population due to the likelihood of co-infection through the common sexual transmission route [57,58]. Co-infection poses a substantial burden on the healthcare system, with estimates of millions of individuals affected [59]. In the United States, the HBV prevalence reported by Abara et al. was up to 17% in PLH compared to 7% in the general population [60]. Buskin et al. also reported a higher prevalence rate for chronic and acute HBV compared to the general population [61]. Moreover, co-infection prevalence is highest in sub-Saharan Africa, ranging from 3.5% to 28% [62]. The disease course associated with this infection profile leads to faster progression to advanced liver disease, including fibrosis, cirrhosis and hepatocellular carcinoma, and increased mortality [63].

PLH and people without HIV have been shown to have a similar response to the standard HBV vaccination if the vaccination is completed prior to the HIV infection [64]. This is not applicable to PLH receiving the HBV vaccine with protection rates as low as 20% [55]. Consequently, numerous studies have been conducted providing evidence on the superior efficacy of a high-dose HBV vaccine compared to the standard dose in PLH [65,66]. In their review article, Whitaker et al. propose multiple strategies to improve HBV vaccine efficacy and durability, including repeat vaccination, the use of double-dose vaccination, an intradermal route of vaccine administration, and the use of adjuvants [67]. Khaimova et al. conducted a retrospective study in which they reviewed 67 patients and determined that 86.6% of HBV vaccine non-responders achieved seroprotection with the adjuvanted Heplislav-B^®^ [68].

### 2.7. Varicella-Zoster

Although there is a paucity of data from clinical trials on the use of varicella vaccines in adolescents and adults infected with HIV, the CDC recommends that PLH who are >8 years old with CD4 counts ≥ 200 cells/mm^3^ would benefit from receiving two doses of Varivax 3 months apart [69].

One million episodes of herpes zoster (HZ) occur yearly in the US according to the CDC [70]. Advancing age represents one of the most important risk factors with a 50% risk of developing HZ over the age of 85 [70,71]. Additionally, at least 85% of the population has experienced prior exposure to VZV. However, the incidence of HZ in PLH is greater than that of the general population [72]. In fact, it has been shown that even after ART initiation, PLH remain at a higher risk of developing HZ than the general population [73]. HIV also increases the risk of recurrence, multi-dermatomal involvement, and progression to systemic disease [74]. Studies have demonstrated a correlation between CD4 counts and the risk of developing HZ in PLH. The CD4 cutoff varies among studies, but a consensus is in favour of a CD4 ≤ 200 cells/mm^3^ being a risk factor of developing HZ in PLH. A low CD4 is also indicative of a likely progression to worse outcomes, specifically when PLH with CD4 counts ≤ 50 cells/mm^3^ develop the disease. One study estimated a cost of 1.2 million Canadian dollars for hospital admissions among 3006 PLH [75].

The VZV vaccine has been shown to provide significant protection again HZ in children living with HIV-1 with an 83% response 1 year after receiving the vaccine. Many clinical trials are currently underway to evaluate the safety and efficacy of the recombinant VZV vaccine (RZV) in PLH with varying CD4 counts.

### 2.8. Human Papilloma Virus

Human Papilloma virus (HPV) remains the most common sexually transmitted infection [76]. High-risk genotypes are associated with malignant neoplasms involving the cervix, oropharynx, and vagina, as well as other cancers. Other low-risk genotypes are more commonly associated with anogenital warts. Vaccination prior to becoming sexually active is key in limiting HPV infection, as well as high-grade squamous epithelial lesions. It is recommended in PLH up to 26 years, as well as MSM and women living with HIV up to 40 years, irrespective of CD4 count, ART use, and viral load.

HIV, even when adequately treated with antiretroviral therapy, predisposes PLH to a higher risk of persistent HPV infection and a faster progression of malignant neoplasms [77,78]. Women with HIV–HPV co-infection are six times more likely to develop cervical cancer [79,80]. In fact, cervical cancer remains one of the most frequent cancers identified in women living with HIV and is currently considered an AIDS-defining illness. Similarly, men with HIV also have a significantly increased risk of anal warts and malignancy due to HPV infection. The CDC estimates that HIV and HPV have the highest healthcare costs out of all sexually transmitted infections, as the treatment is life-long and the patient is at risk of malignant neoplasms. The total healthcare cost for HIV infections was estimated at USD 13.7 billion, while USD 755 million dollars were attributed to HPV infections [81]. HPV vaccine uptake in PLH remains limited [82], despite the proven efficacy and safety of the HPV vaccine in individuals with HIV [78,83].

Few studies have addressed vaccine efficacy in PLH. HPV vaccine efficacy has been reported as above 90% in multiple studies [84,85]. However, high HIV RNA levels (>10,000 copies/mL) and CD4 < 200 cells/mm^3^ resulted in a less favourable vaccine response [86].

### 2.9. Measles, Mumps, Rubella

PLH are at an increased risk of severe measles infection. Adults living with HIV are likely to be immune against measles (90% with detectable antibodies) [87]. If indicated, and since MMR is a live attenuated vaccine, it is given to PLH with a CD4 cell count ≧200 cells/mm [39]. Initial vaccination should contain two doses, with a potential third dose given 2–5 years after primary vaccination if the CD4 cell count is above 200 cells/mm [88]. In 80% of immunocompromised patients, measles led to severe complications, with 58% presenting pneumonitis, compared to 1–6% in immunocompetent hosts [89]. The fatality rate of measles pneumonitis is estimated between 33 and 45% in PLH [89]. PLH are disproportionately affected by mumps, representing 17% of cases of mumps in a study in Chicago, while only representing <1% of the population [90].

Immunity to MMR wanes over time; in a cross-sectional study, 26 PLH with CD4 above 200 cells/mm compared to 22 controls were given the MMR vaccine [91]. One year post vaccination, a higher proportion (57%) of PLH lost measles antibodies compared to the controls (11%); the cellular responses were similar between groups. The improvement of seroprotection has been shown for measles if HAART is given within 1 year of immunization [92].

### 2.10. Mpox

The CDC recommends vaccination through two doses of JYNNEOS vaccine for effective protection against mpox [93].

For PLH, and other immunocompromising conditions, mpox carries significant morbidity and mortality. In a cohort of 382 mpox cases, those with immunocompromising conditions had increased skin necrosis (54% to 7%), sepsis (44% to 9%), and even death (only in those with immunosuppression) [94]. As of 2023, 157 deaths have been reported in PLH with mpox [95]. Forty percent of US mpox patients are PLH [96].

While Morales et al. report a vaccine effectiveness of 88% when given as post-exposure prophylaxis (PEP) [97], Rosen et al. demonstrate the reasons why it is difficult to obtain an accurate estimate of PEP vaccine efficacy [98]. A delay in PEP receipt is generally present after exposure to mpox, with an average 7-day delay from exposure to vaccination according to the authors. This was largely due to delay in seeking care or in receiving a notification of exposure to the disease. If the incubation period for mpox was close to the delay in PEP administration, patients exhibiting symptoms were no longer eligible for PEP. This model likely overestimated PEP efficacy [98]. The CDC recommends administering a single dose of the vaccine as PEP within the first four days of exposure, with unclear but suspected benefit if administered within 14 days [99]. Like MMR, for PLH, a CD4 cell count ≥ 200 cells/mm is needed for safe vaccination against mpox [100]. Peak immunity for JYNNEOS vaccination is 14 days, but there is currently no information on the duration of immunity for PLH [100].

### 2.11. Tetanus, Diphtheria, Pertussis

It is recommended that Tdap vaccination be administered to all PLH who have not received primary vaccination series for tetanus, diphtheria, or pertussis [27]. This should be followed by Tdap or TD vaccinations at 4 weeks and 6–12 months after initial vaccination. There is also a recommendation for a booster of tetanus–diphtheria (TD) or Tdap every 10 years for PLH [39].

For PLH, tetanus can lead to devastating mortality rates. In a cohort of 21 patients with tetanus, half were PLH who had a significantly higher mortality rate of 81% compared to non-immunocompromised patients (12%) [101]. The increased risk of tetanus disease acquisition in PLH needs to be further studied. While there is no current study on Tdap vaccine immunogenicity over time, similar antibody levels post vaccination for PLH and those without have been reported. However, protective antibodies for diphtheria in PLH have shown a decrease, with antibody levels of 83–100% and 61–73% for individuals without HIV and PLH, respectively [39].

### 2.12. Respiratory Syncytial Virus

Respiratory syncytial virus (RSV) vaccination has recently been approved by the ACIP based on shared decision making for adults aged 60 and older, including PLH [102]. The current recommendation is one RSV vaccination dose.

There are minimal data on the epidemiology of RSV in the US in PLH. The rate of severe symptoms from RSV symptoms is usually influenced by underlying medical conditions, with 17% of hospitalized RSV patients requiring ICU admission, with 5% dying [103]. Among immunocompromised individuals, the mortality rate has been as high as 60% [104].

Additionally, the evidence on vaccine efficacy remains limited [105].

### 2.13. SARS-CoV-2

At the start of the COVID-19 pandemic, there were concerns over the increased risk of infection for immunocompromised people, such as those with HIV. The CDC currently recommends that all PLH, regardless of CD4 count, receive the two-dose primary series of mRNA COVID-19 vaccination given 3–8 weeks apart, followed by a booster vaccination dose as recommended by the Advisory Committee on Immunization Practices (ACIP) [106].

PLH had a significantly higher risk of SARS-CoV-2 infection [107]. PLH are also at a greater risk for death from COVID-19 [108]. There is a demographic disparity within PLH and a greater mortality risk observed in African Americans compared to other races [109].

Vaccine coverage for the primary series was lower for PLH—78.2% versus 81.8% for people without HIV. However, the first booster coverage was higher for PLH—68.5% compared to those without (63.1%) [110].

Some studies demonstrated decreased vaccine effectiveness in PLH. SB. Coburn et al. included 31,840 PLH and 77,759 participants without HIV. The breakthrough infection incidence rate at 210 days post full vaccination was 2.8% [2.6%, 3.1%] for PLH compared to 2.1% [2.0% 2.3%] for participants without HIV, with a *p*-value < 0.01 [111].

## 3. Burden of Vaccine-Preventable Diseases in PLH

HIV is associated with a high incidence of VPDs [112]. Table 1 provides a summary of the reported incidence or prevalence rates of common VPDs in PLH.

Healthcare costs for the management of PLH is significantly higher than the general population [113]. This represents the cost of treatment of HIV and VPDs but also the cost of loss in quality of life, missed workdays, physician appointments, and hospitalizations. In fact, the cost of healthcare per patient was six times greater in PLH compared to people not living with HIV and four times greater when excluding the cost of ART treatment [113]. Liver disease due to hepatitis was also a main culprit in increased healthcare costs in PLH in California [114]. While an extensive deep dive on the economic burden of VPDs in PLH has not been undertaken yet, it is reasonable to presume that combining the cost of treatment of HIV with the treatment of VPDs undoubtedly imposes additional financial stress on the healthcare system.

The stigmatization around HIV-infected individuals still represents the biggest societal burden related to VPDs in PLH [115]. Stigma often derives from lack of knowledge and can be seen with other VPDs in the presence or absence of HIV co-infection [116,117]. Studies have proved a significant association between HIV and mental health disorders largely due to stigmatization and isolation. Major depression and anxiety were the two mood disorders most frequently diagnosed in PLH [118,119].

**Table 1:** VPD disease burden and vaccination rates in PLH.

Vaccine-Preventable Disease	Prevalence in PLH	Vaccination Rate
Influenza	46 (PLH) vs. 15 (non-PLH) per 1000 person years [120]	50% higher vaccination rate compared to general population [15]
Pneumococcal disease	160 (PLH) vs. 8 (non-PLH) per 100,000 person years [121]	64.6% [122]
Hepatitis B	Co-infection rate of 8–10% [123] 11.4% (Western pacific region); 10% (sub-Saharan Africa); 6.7% (Europe); 5.3% (America) [123]	61.9% received at least one vaccination dose from the series [122]
Herpes Zoster	101 per 100,000 person years [72]	More data needed
HPV	40.6% (PLH) vs. 21.4% (non-PLH) [124]	HPV vaccine acceptance rate of 59.19% [125]

## 4. Barriers to Vaccine Uptake in PLH

Several challenges are identified and need to be surpassed when considering vaccination uptake in vulnerable populations such PLH.

First, the diminished immune system in PLH leads to a faster decrease in seroprotection provided by available vaccines [92]. This reduced response has been studied in many vaccines such as Hepatitis B [55] and more recently extensively studied for the SARS-CoV-2 Vaccine [126,127]. In fact, the lower the CD4+ count, the lower the immunogenicity of the vaccine administered, especially with CD4+ counts of less than 200 [128]. Therefore, the need for multiple boosters, use of intradermal delivery route, higher vaccine doses, or the addition of adjuvants might make adherence to a strict vaccine schedule more complicated and worsen vaccine hesitancy.

Second, there is a gap in the knowledge on this topic, both among healthcare workers and PLH. VPDs are reported to be more serious in PLH with lower CD4+ counts; a more invasive pneumococcal disease, higher mortality related to influenza, and even higher prevalence of meningococcal disease have been reported across studies [129,130]. These findings align with other reports of lower vaccine uptakes for those diseases in all populations but more particularly in PLH [131]. One possible cause is misinformation regarding the safety and efficacy of said vaccines, which leads to patients refusing vaccination. If the information the physician provides is not clear on indication, potential benefits, and risks, vaccine hesitancy can increase significantly [129]. In recent years, and as highlighted by the COVID-19 pandemic, growing concerns over vaccine safety also adds to the barriers to vaccination in PLH. PLH seem to be increasingly concerned with the potential harm vaccination could cause to their health [132]. Furthermore, the presence of misinformation in the media about vaccine safety hinders vaccination in PLH, especially those with lower levels of education and older age [132]. Of note, PLH with CD4 counts ≥ 200 cells/mm^3^ were more likely to be vaccinated against COVID-19 compared to PLH with lower CD4 counts [133].

Third, structural barriers also need to be considered when attempting optimal vaccination status in PLH. When multiple booster doses are required in the PLH population, multiple visits to the physician’s office are therefore needed [134]. Table 2 highlights vaccination milestone recommendations in PLH. In lower socio-economic environments, this could pose a serious threat to a complete vaccination status. Moreover, it is quite frequent to have multiple people involved in the care of PLH. Therefore, having a multi-disciplinary team with excellent communication remains key to achieving full and comprehensive vaccination in those populations [129].

Stigma and discrimination remain a major source of reduced vaccine uptake in PLH. First, stigma and discrimination lead to discouragement in seeking healthcare and health services in general [135], hence lowering access to a full vaccination schedule. With internalized stigma and discrimination comes increased vaccine hesitancy and uptake [136]. This vicious cycle poses a serious threat to not only vaccine uptake but also access to care in general in the PLH population.

**Table 2:** Vaccination recommendation in PLH according to the IDSA (2013) and British HIV Association (2015).

	IDSA [5]	British HIV Association [3]
PCV13	HIV-infected patients aged ≥ 2 years	Single dose irrespective of CD4 count, ART use, and viral load
PPSV23	(a) HIV-infected children aged ≥ 2 years of age who have received indicated doses of PCV (b) HIV-infected adults with CD4 T-lymphocyte counts of ≥200 cells/mm^3^ (c) HIV-infected adults with CD4 T-lymphocyte counts of <200 cells/mm^3^ (d) Given ≥8 weeks after indicated dose(s) of PCV13, and a second dose of PPSV23 should be given 5 years later	(a) General guidance on PPSV23 (b) At least 3 months between PCV13 and PPSV23
Hib	Not recommended for HIV-infected adults	(a) Not routinely recommended (b) Recommended in HIV-positive adults with asplenia, splenic dysfunction or complement deficiency
HBV	High-dose HBV vaccine for adults and adolescents If post-vaccination concentration is not ≥10 mIU/mL, a second three-dose series of HBV is recommended	(a) High-dose vaccination for yeast-based vaccine (b) Regular-dose vaccination for adjuvanted vaccine (c) Four vaccine doses at 0, 1, 2, 6 months (d) Ultra-rapid vaccination within 3 weeks in patients with CD4 > 500 cells/mm^3^ if full vaccination is required urgently or if compliance with full course is doubtful (e) High-dose vaccination not recommended in ultra-rapid schedule (f) If post-vaccination concentration is not ≥10 mIU/mL, a second three-dose series of HBV is recommended at monthly intervals (g) Revaccination with regular dose vaccine preferred
HPV	HPV4 is preferred over HPV2 (The 9vHPV is the vaccine currently recommended)	(a) Vaccination recommended in HIV-positive individuals aged up to 26 years irrespective of CD4 count, ART use, and viral load (b) Unvaccinated MSM living with HIV and aged up to 40 years irrespective of CD4 count, ART use, and viral load (c) Unvaccinated women living with HIV aged up to 40 years irrespective of CD4 count, ART use, and viral load (d) Defer vaccination in ART naïve HIV patients with CD4 < 200 cells/mm^3^ (e) 3 doses of 4vHPV on 0, 1, 2 months, and 6 months. (f) PLH who have received < 3 doses of 4vHPV before age 18 should complete the three-dose course. (g) The 9vHPV is the preferred vaccine (h) HPV vaccination to be considered in HIV-positive patients with high-grade HPV disease to reduce risk of recurrence
Influenza	Annual inactivated influenza vaccine recommended in PLH including pregnant women	Annual inactivated influenza vaccine recommended in PLH, including pregnant women
MMR	(a) Recommended in HIV-infected children aged between 1 and 13 years without severe immunosuppression (b) Recommended in HIV-infected individuals aged over 14, non-immune to measles, and with a CD4 count ≥ 200/mm^3^ (c) Not recommended in children with CD4 T-cell percentage < 15 or patients aged > 14 with CD4 count < 200 cell/mm^3^ (d) Quadrivalent MMR-varicella not recommended	(a) Two doses, at least 1 month apart, recommended in HIV-infected individuals non-immune to measles and a CD4 count ≥ 200/mm^3^ (b) Recommended in HIV-positive non-pregnant women of child-bearing age who are Rubella seronegative and have a CD4 count > 200 cells/mm^3^
Varicella	(a) Recommended in children 1–8 years, stable, with ≥15 CD4 T-lymphocyte percentage (b) 9–13 years with ≥15 CD4 T-lymphocyte percentage (c) Aged ≥ 14 years with CD4 T-lymphocyte counts ≥ 200 cells/mm^3^ (d) Two doses recommended separated by ≥3 months	(a) Two doses 3 months apart recommended in seronegative HIV-positive adults with CD4 T-lymphocyte counts ≥ 200 cells/mm^3^ and established on ART (b) One dose recommended in seropositive HIV-positive adults with CD4 T-lymphocyte counts ≥ 200 cells/mm^3^ and established on ART

## 5. Strategies for Improving Vaccine Uptake

Vaccine uptake in PLH is inconsistent at best and requires serious effort to implement evidence-based strategies for vaccination. The CDC proposes the 4 Pillars™ Practice Transformation Program that represents a strong attempt at implementing a standardized approach to increasing vaccine uptake among vulnerable patients [137]. The four pillars focus on making vaccines more convenient in the clinic, discussing vaccination importance with patients, enhancing systems to facilitate vaccination, and increasing motivation. Nowalk et al. demonstrated the efficacy of this program in primary care clinics to encourage vaccination in high-risk adults. This program is a framework that aims to make vaccines more accessible, promote patient education, enhance systems promoting vaccinations, and increase motivation, with the goal of achieving higher vaccination rates. Wells et al. utilized this tool and tailored it specifically towards increasing HPV vaccination in PLH [138]. SARS-CoV-2 vaccination rates were shown to be higher in PLH over the age of 40 and who had previously received the influenza vaccine. Vaccine trust and the fear of developing severe COVID-19 were associated with improved vaccine uptake. However, factors such as low education levels and unemployment were associated with low vaccine uptake [139]. When attempting to draw lessons from strategies in this study, it seems that improving knowledge on various VPDs and educating PLH on vaccine efficacy in clinics could address the vaccine hesitancy issue and undoubtedly improve vaccine uptake among PLH. Johnson et al. demonstrated that a high rate of missed appointments (>10%) was associated with lower vaccination rates, as these patients had less opportunities for exposure to vaccines and vaccine education. MSM also had lower vaccination rates among PLH, further emphasizing the necessity to implement strategies limiting health disparities in MSM and other vulnerable populations.

## 6. Conclusions

In conclusion, VPDs still pose a major health concern in PLH as either a failure of vaccines or, mostly, a failure to vaccinate. While ART has been ground breaking in the management of PLH, it has not led to equal immunogenicity, efficacy, or effectiveness to vaccines comparable to those in the general population. Vaccines represent a pivotal tool in safeguarding the health of this vulnerable population. However, vaccination rates remain subpar, highlighting the need for targeted vaccination strategies in PLH.
